# CD4 Counts and Viral Loads of Newly Diagnosed HIV-Infected Individuals: Implications for Treatment as Prevention

**DOI:** 10.1371/journal.pone.0090754

**Published:** 2014-03-04

**Authors:** Sarishen Govender, Kennedy Otwombe, Thandekile Essien, Ravindre Panchia, Guy de Bruyn, Lerato Mohapi, Glenda Gray, Neil Martinson

**Affiliations:** 1 Perinatal HIV Research Unit (PHRU), University of the Witwatersrand and Baragwanath Hospital, Soweto, South Africa; 2 Johns Hopkins University, Center for TB Research, Baltimore, Maryland, United States of America; 3 Canada-Africa Prevention Trials Network, The Ottawa Hospital General Campus, Ottawa, Canada; Institute of Infection and Global Health, United Kingdom

## Abstract

**Objective:**

To report the viral load and CD4 count in HIV-infected, antiretroviral naïve, first -time HIV-testers, not immediately eligible for treatment initiation by current South Africa treatment guidelines.

**Design:**

This was a cross-sectional study in a high-volume, free-of-charge HIV testing centre in Soweto, South Africa.

**Methods:**

We enrolled first time HIV testers and collected demographic and risk-behaviour data and measured CD4 count and viral load.

**Results:**

Between March and October 2011, a total of 4793 adults attended VCT and 1062 (22%) tested positive. Of the 1062, 799 (75%) were ART naïve and 348/799 (44%) were first-time HIV testers. Of this group of 348, 225 (65%) were female. Overall their median age, CD4 count and viral load was 34 years (IQR: 28-41), 364 (IQR: 238-542) cells/mm^3^ and 13,000 (IQR: 2050-98171) copies/ml, respectively. Female first time HIV testers had higher CD4 counts (419 IQR: 262-582 vs. 303 IQR: 199-418 cells/mm^3^) and lower viral loads (9,100 vs. 34,000 copies/ml) compared to males. Of 183 participants with CD4 count >350 cells/mm^3^, 62 (34%) had viral loads > 10,000 copies/ml.

**Conclusions:**

A large proportion of HIV infected adults not qualifying for immediate ART at the CD4 count threshold of 350 cells/mm^3^ have high viral loads. HIV-infected men at their first HIV diagnosis are more likely to have lower CD4 counts and higher viral loads than women.

## Introduction

Anti-retroviral therapy (ART) initiation has been shown to dramatically reduce HIV transmission in discordant heterosexual couples prompting revisions to treatment eligibility criteria. [1]–[3] Responding to this, new guidelines recommend starting ART either at HIV diagnosis, or at CD4 counts of ≤500 cells/mm^3^. [4], [5] Current South African (SA) treatment guidelines for ART include recommendations for treatment initiation at a CD4 threshold of ≤ 350 cells/mm^3^ in non-pregnant, well adolescents and adults.[4], [5].

ART initiation is traditionally based on CD4 counts. In conjunction with viral loads, they allow prognostication. [6] Moreover, viral loads provide a measure of infectivity; individuals with low or suppressed viral loads have markedly lower transmission rates. [7], [8]

This study reports the distribution of CD4 counts and viral loads in ART-naïve, first-time HIV testers and relates CD4 counts to current South African ART initiation thresholds, and the proportion of participants not qualifying for immediate initiation of ART by the CD4 criterion but who have high viral loads and high potential of onward HIV transmission.

## Materials and Methods

We analysed data collected at the ZAZI clinic which is a free-of-charge, voluntary counselling and testing (VCT) facility on the campus of the Chris Hani Baragwanath Academic Hospital in Soweto, South Africa. The clinic is connected by a pedestrian bridge to a busy taxi rank. ZAZI tested 15,824 walk-in patients from October 2010 to September 2011. Participants included in this analysis had to be ART-naïve, self-report being HIV tested for the first time, and have no clinical evidence of an AIDS defining condition (WHO Stage 1 or 2 diseases).

### Study design

This was a cross-sectional study. All consenting participants were sequentially enrolled, had demographic and behavioural profiles captured and were symptom-screened for tuberculosis and other opportunistic infections. Behavioural profile questions related to number of sexual partners, condom and alcohol usage in the month prior to testing were collected using face-to-face interviews in routine VCT counselling procedure by counsellors. Blood draws for CD4 count and viral load were taken at the time of HIV diagnosis. Those eligible for ART (with CD4 ≤ 350 cells/mm^3^ as per South African HIV treatment guidelines [4]) were referred to treatment facilities whereas the remaining were followed up at ZAZI at six month intervals. Study data collection was conducted between March 2011 and October 2011.

HIV testing was by double-rapid test algorithm. Those testing negative on SD Bioline HIV 1/2 3.0 rapid test (Standard Diagnostics Inc, Gyeonggi-do, Republic of Korea) were not retested. Positive rapid tests were confirmed with a second rapid test (First Response, Premier Medical Corporation Limited, Daman, India). Discordant rapid test results were resolved with a laboratory HIV ELISA test. Blood was drawn and stored in tubes in room temperature where laboratory staff would collect them daily at scheduled times. No blood stayed overnight in the clinic. HIV-infected clients received CD4 and viral load results within two days of testing and those eligible for treatment and care were referred to local clinics.

### Laboratory testing

Viral Load testing was conducted using the Nuclisens Nucleic Acid Sequence Based Amplification (NASBA, bioMerieux, Inc. Durham, NC) test platform for most tests (the detection limit for HIV viral load was < 400 copies/ml) and the Real Time HIV-1 Amplification Polymerase Chain reaction (PCR) (ABBOTT Molecular, Inc, Des Plaines, IL) platform in a few instances. CD4 cells were enumerated using the dual platform method. [9].

Written consent was obtained from each participant for their information to be stored in the clinic database and used for research purposes. The study was approved by the University of the Witwatersrand Human Research Ethics Committee.

### Statistical analysis

Median and interquartile ranges were determined for age, CD4 count and viral loads. Continuous measures were compared using the Kruskall-Wallis non-parametric test. CD4 count was categorised dichotomously based on the South African treatment guidelines while viral loads were classified using >10,000 copies/ml as probable high infectivity, based on prior HIV transmission related studies.[8], [10], [11] Overall frequencies and their percentages were determined for categorical variables and by gender. The viral loads between males and females were compared while adjusting for CD4 count and age using analysis of covariance. Behavioural variables stratified by CD4 count and viral load categories were compared using the chi-square test. We determined the correlation between log_10_ viral load versus CD4 count, and included the least squares line by gender on a scattergram. All hypothesis testing used two-sided tests at 5% significance level using SAS 9.3 (SAS Institute, Cary, NC) statistical software.

## Results

**Figure 1:**
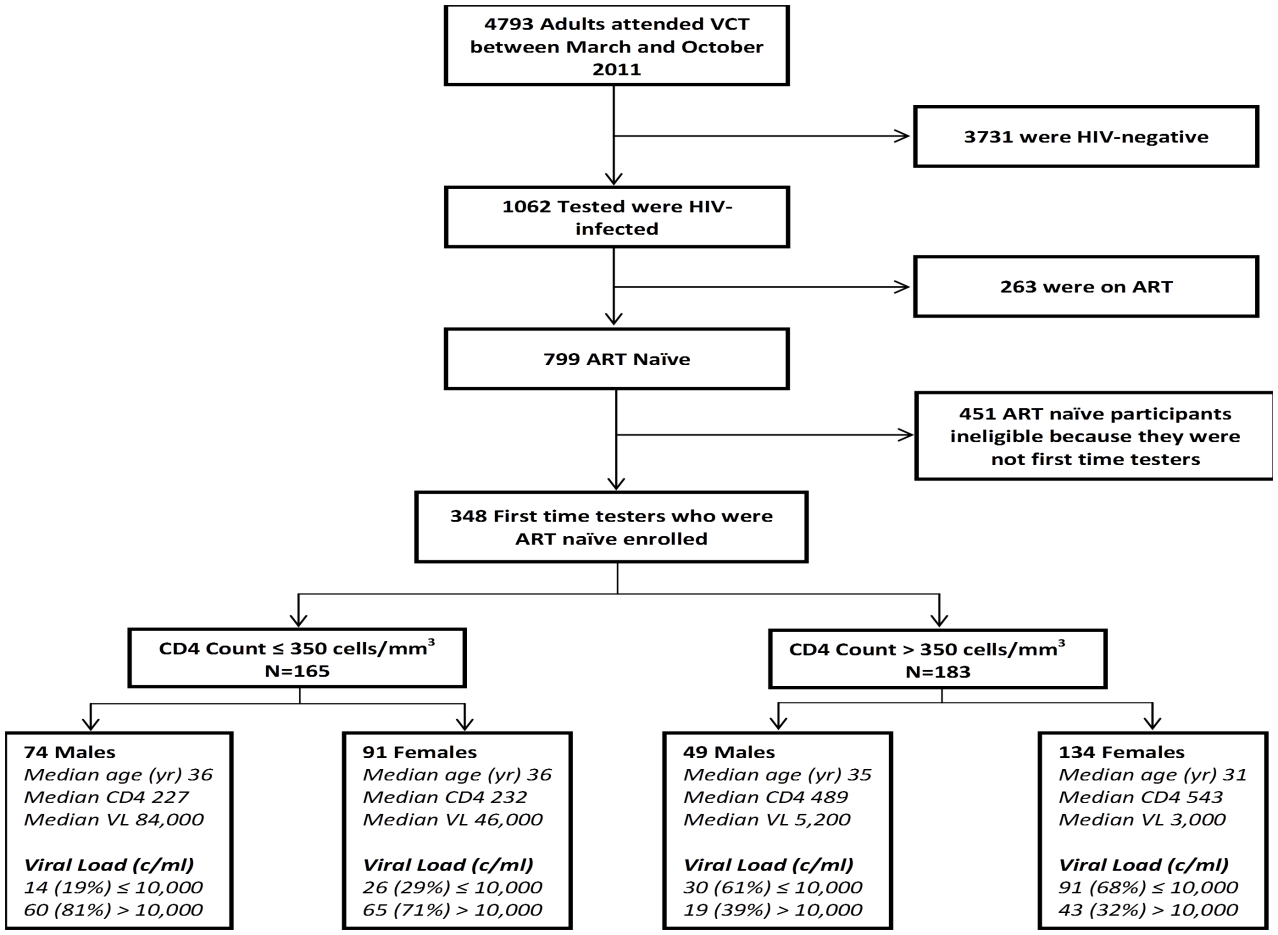
Participant Disposition Flow Diagram.

A total of 4,793 adults attended VCT between March and October 2011, of whom 1062 (22%) tested HIV-positive (Figure 1). Of the 1062 HIV-infected adults, 799 (75%) were ART naïve, of whom 348 (44%) were first time testers. The leading reasons for exclusion of ineligible participants were: being previously tested (n = 451) and not being ART naïve (n = 263). The median age of all ineligible participants was 35 years (IQR: 30-40), their median CD4 count and viral load was 304 cells/mm^3^ (IQR: 188-446) and 26,000 copies/ml (IQR: 4,100-140,000), respectively. Ineligible participants were excluded from further analyses.

The median age of the ART-naïve, first time testers was 34 years (IQR: 28-41). Males presented at older age than females (36 vs. 33; p = 0.0086). The overall median CD4 count was 364 cells/mm^3^ (IQR: 238.0-541.5); median CD4 count in females was higher than males (419 cells/mm^3^ vs. 303 cells/mm^3^; p = 0.0001). A total of 183 (53%) participants had CD4 count >350 cells/mm^3^; the proportion of females with CD4 above 350 cells/mm^3^ was higher than males (60% vs. 40%, p = 0.0004; Table 1).

**Table 1:** Characteristics of all asyptomatic, first time HIV testers at enrolment.

Variable	Overall(N = 348)	Male(N = 123)	Female(N = 225)	p-value
**Median Age at enrolment (IQR)**	34 (28–41)	36 (30–42)	33 (27–40)	0.0086
**Median CD4 Count (IQR)**	364 (238–542)	303 (199–418)	419 (262–582)	0.0001
**CD4 Count Categories**				
≤ 350 (%)	165 (47.4)	74 (60.2)	91 (40.4)	0.0004
> 350 (%)	183 (52.6)	49 (39.8)	134 (59.6)	
**Median Viral Load (IQR)**	13000	34000	9100	0.0005
	(2050-98171)	(2900-210000)	(1500-62000)	
**Viral Load Categories**				
≤ 10,000 c/ml (%)	161 (46.3)	44 (35.8)	117 (52.0)	0.0037
> 10,000 c/ml (%)	187 (53.7)	79 (64.2)	108 (48.0)	
**No. of partners**				
≤ 1 (%)	286 (90.8)	97 (82.2)	189 (95.9)	<0.0001
>1 (%)	29 (9.2)	21 (17.8)	8 (4.1)	
**Condom usage** *				
No (%)	113 (36.0)	43 (36.8)	70 (35.5)	-
Not sexually active (%)	7 (2.2)	1 (0.9)	6 (3.1)	
Sometimes (%)	15 (4.8)	7 (6.0)	8 (4.1)	
Yes (%)	179 (57.0)	66 (56.4)	113 (57.4)	
**Alcohol usage** *				
Yes (%)	159 (50.5)	84 (71.2)	75 (38.1)	<0.0001
No (%)	156 (49.5)	34 (28.8)	122 (61.9)	
**Reason for testing**				
General check-up (%)	272 (78.4)	110 (90.2)	162 (72.0)	-
Illness (%)	13 (3.8)	6 (4.9)	7 (3.1)	
Pregnancy (%)	13 (3.8)	N/A	13 (5.8)	
Unknown (%)	33 (9.5)	5 (4.1)	28 (12.4)	
Other (%)	16 (4.6)	1 (0.8)	15 (6.7)	

* In the last month prior to the study.

P-values for condom usage and reason for testing not presented due to small numbers within group.

Overall the median viral load for ART-naïve, first time testers was 13,000 copies/ml (IQR: 2050-98,171). Females had a markedly lower median viral load compared to males (9,100 vs. 34,000 copies/ml, p = 0.0005). Overall, 187 (54%) participants had viral loads > 10,000 copies/ml. The proportion of males with viral loads >10,000 copies/ml was significantly higher than females (64% vs. 48%; p = 0.0037). Of the 183 participants with CD4 count >350 cells/mm^3^, 62 (34%) had a viral load > 10,000 copies/ml.

The proportion of males and females with CD4 count >350 cells/mm^3^ and with viral loads >10,000 copies/ml were similar (males 19/49 [39%] vs. females 43/134 [32%]; p = 0.3975). The distribution of CD4 counts within the two viral load strata (> 10,000 and ≤ 10,000 copies/ml) was not statistically different (Table 2). After adjusting for CD4 count and age, females had a significantly lower HIV viral load compared to males (p = 0.0067).

**Table 2:** Distribution of viral loads by CD4 count categories above 350 cells/mm.

CD4 CountCategory	Median (IQR)	≤ 10,000 copies/ml (N = 121)	> 10,000 copies/ml (N = 62)	p-value *
350–399	8,400 (2,000–36,000)	15 (12%)	14 (23%)	0.0742
400–449	3,000 (280–14,000)	24 (20%)	9 (15%)	0.3758
450–499	5,700 (1,100–30,000)	13 (11%)	6 (10%)	0.8229
≥ 500	2,800 (360–15,000)	69 (57%)	33 (52%)	0.6243

*p-value represents the comparison of proportions with viral loads below and above 10,000 copies/ml.

**Figure 2:**
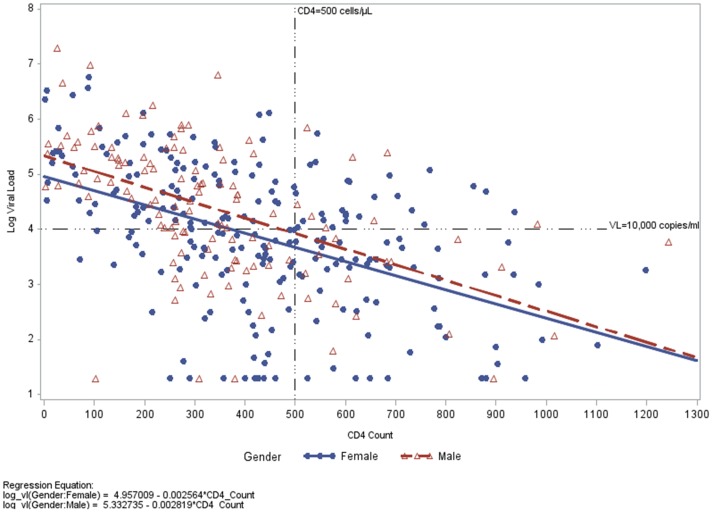
Correlation plot of log_10_ viral load and CD4 count taken at the same visit, stratified by gender with least-squares line for males and females.

Of those with CD4 > 350 cells/mm^3^ and VL > 10,000 copies/ml, males were more likely to report alcohol use (15/19 (79%) vs. 13/39 (33%), p = 0.0011). Those with a CD4 ≤ 350 cells/mm^3^ were more likely to have more than one partner than participants with CD4 > 350 (p = 0.0414). Condom use, alcohol use and reason for HIV testing were similar between the two CD4 strata of ≤ 350 and > 350 cells/mm^3^ (Table 3). There was a negative moderate Pearson correlation of -0.504 between log_10_ viral load and CD4 count (Figure 2) for all participants.

**Table 3:** Distribution of behavioural characteristics by CD4 and viral load.

Variable	CD4 ≤ 350	CD4 > 350	p-value *	Viral Load ≤ 10,000	Viral Load > 10,000	p-value *
**Number of partners**						
>1 (%)	19/165 (13.0)	10/183 (5.9)	0.0414	10/161 (6.8)	19/187 (11.2)	0.1838
**Condom usage**						
Yes (%)	82/165 (56.2)	97/183 (57.7)	0.5375	86/161 (59.3)	93/187 (55.0)	0.4930
**Alcohol usage**						
Yes (%)	79/165 (54.1)	80/183 (47.3)	0.4363	69/161 (47.3)	90/187 (53.3)	0.3250
**Reason for testing**						
General check-up (%)	131/165 (79.4)	141/183 (77.5)	0.5971	121/161 (75.2)	151/187 (81.2)	0.2079

*p-value represents the comparison of proportions between CD4 count and viral load categories.

## Discussion

We report CD4 counts and viral loads in adult first-time HIV testers who were well, ART naïve, and diagnosed with HIV at an easily accessible, high volume, free-of-charge VCT centre. A large proportion of HIV infected adults not qualifying for immediate ART at the CD4 count threshold of 350 cells/mm^3^ had high viral loads. Of the ART-naïve first time testers whose CD4 count was above the CD4 threshold for ART initiation as per South African guidelines, 34% had a VL > 10,000 copies/ml suggesting that CD4 count at the time of HIV diagnosis is a poor proxy for HIV transmission risk. Consideration should be given to replacing CD4 count threshold with viral load threshold for ART initiation when planning treatment as prevention (TasP) interventions.

Our results show that most asymptomatic adults requesting HIV testing for the first time had low CD4 counts (median CD4 count 364 cells/mm^3^) and/or high viral loads (median viral load 13,000 copies/ml). Indeed, almost half of all the asymptomatic first-time testers in this study qualified for immediate initiation of ART according to current South Africa guidelines; most were men. This result underlies the importance of reducing the time to first testing in men. Other studies have noted gender differences in viral loads and CD4 at various stages of the disease, where females develop AIDS at higher CD4 counts and lower viral loads.[12]–[15] The higher median viral load of males increases the rate of disease progression, CD4 decline and relative infectivity (when in a discordant relationship).[1], [16].

Limitations to this study include social desirability bias that may have resulted in misclassification of participants as first-time testers and being ART-naïve. Moreover, the cross- sectional nature of the study does not reflect the dynamic nature of CD4 counts.

Although not an original part of this research, a surprising finding was the high number of HIV testers who were taking ART. They were identified by their low viral loads, and on further questioning reported receiving ART. We speculate, in the absence of supporting data, that there is a sizable group of patients on ART who either want a CD4 count without having to undergo a formal ART clinic visit, or want to confirm their HIV-infection after being told in lay terms that their HIV (viral load) is undetectable.

TasP requires population screening approaches to identify HIV-infected people at the earliest possible opportunity and start them on suppressive antiretroviral therapy to prevent further transmission. This potentially optimal HIV-testing setting (free-of-charge, easily accessible, short waiting times) identified HIV-infected individuals with high viral loads and relatively low CD4 counts. More research is needed to encourage sexually active adults to cyclically present themselves for HIV testing. Moreover, viral load estimation at the time of HIV diagnosis might be a better measure than CD4 count when making decisions about initiation of ART in the context of TaSP. Our data suggest that for TasP to succeed, ART treatment initiation criteria require revision, with a view to commencing therapy irrespective of CD4 counts.
